# Cardiovascular Risk Factors Increase the Risks of Diabetic Peripheral Neuropathy in Patients With Type 2 Diabetes Mellitus: The Taiwan Diabetes Study

**DOI:** 10.1097/MD.0000000000001783

**Published:** 2015-10-23

**Authors:** Chun-Pai Yang, Cheng-Chieh Lin, Chia-Ing Li, Chiu-Shong Liu, Wen-Yuan Lin, Kai-Lin Hwang, Sing-Yu Yang, Hsuan-Ju Chen, Tsai-Chung Li

**Affiliations:** From the Department of Neurology, Kuang Tien General Hospital (C-PY); Department of Nutrition, Huang-Kuang University (C-PY); School of Medicine, College of Medicine, China Medical University (C-CL, C-IL, C-SL, W-YL); Department of Medical Research (C-CL, C-IL, C-SL); Department of Family Medicine, China Medical University Hospital (C-CL, C-SL, W-YL); Department of Public Health, Chung Shan Medical University (K-LH); Graduate Institute of Biostatistics, College of Public Health, China Medical University (S-YY, T-CL); Management Office for Health Data, China Medical University Hospital (H-JC); and Department of Healthcare Administration, College of Medical and Health Science, Asia University, Taichung, Taiwan (T-CL).

## Abstract

This study aimed to examine whether poor glycemic control, measured by glycated hemoglobin A1C (HbA1c) and other cardiovascular risk factors, can predict diabetic peripheral neuropathy (DPN) in patients with type 2 diabetes mellitus (DM).

Patients aged ≥30 years with type 2 DM, enrolled in the National Diabetes Care Management Program, and free of DPN (n = 37,375) in the period 2002 to 2004 were included and followed up until 2011. The related factors were analyzed using Cox proportional hazards regression models.

For an average follow-up of 7.00 years, 8379 cases of DPN were identified, with a crude incidence rate of 32.04/1000 person-years. After multivariate adjustment, patients with HbA1c levels 7 to 8%, 8 to 9%, 9 to 10%, and ≥10% exhibited higher risk of DPN (adjusted HR: 1.11 [1.04–1.20], 1.30 [1.21–1.40], 1.32 [1.22–1.43], and 1.62 [1.51–1.74], respectively) compared with patients with HbA1c level 6 to 7%. There was a significant linear trend in DPN incidence with increasing HbA1c (*P* < 0.001) and significant HRs of DPN for patients with HbA1c level ≥7%, blood pressure ≥130/85 mm Hg, triglycerides (TG) ≥150 mg/dL, high density of lipoprotein-cholesterol (HDL-C) <40 mg/dL in males and <50 mg/dL in females, low density of lipoprotein-cholesterol (LDL-C) ≥100 mg/dL, and estimated glomerular filtration rate (eGFR) <60 mL/min/1.73 m^2^.

Patients with type 2 DM and HbA1c ≥7.0% exhibit increased risk of DPN, demonstrating a linear relationship. The incidence of DPN is also associated with poor glucose control and cardiovascular risk factors like hypertension, hyper-triglyceridemia, low HDL-C, high LDL-C, and decreased eGFR.

## INTRODUCTION

Diabetic peripheral neuropathy (DPN) is associated with considerable morbidity, increased mortality, and diminished quality of life, causing a tremendous economic burden in patients with type 2 diabetes mellitus (DM).^1–3^ Its lifetime prevalence in type 2 DM may be up to 50%.^4^ Multiple trials have demonstrated that enhanced glucose control alone is not enough to prevent the onset and progression of DPN in type 2 DM.^5,6^ Thus, the identification of new modifiable risk factors is essential.

Recently, cardiovascular risk factors like obesity, dyslipidemia, and hypertension have been associated with DPN in patients with type 2 DM.^7–11^ Identifying modifiable risk factors may lead to new risk reduction strategies. Four studies have adopted a cross-sectional study design^7–10^ and 1 is a randomized trial with the primary aim of exploring whether triglyceride level is associated with the progression of diabetic neuropathy in 217 patients with type 2 DM during a 52-week follow-up,^11^ instead of examining the association between triglyceride level and DPN incidence. Furthermore, most of these studies have a small sample size.^7,9,11^ Whether potential cardiovascular risk factors are associated with increased risk of DPN has not been explored in large-scale studies.

This large retrospective cohort study aimed to examine whether poor glycemic control, measured by HbA1c level, and other cardiovascular risk factors have a significant independent association with DPN in patients with type 2 DM. The interaction and joint association between HbA_1_c level and these cardiovascular risk factors were also examined.

## METHODS

### Study Population

This retrospective cohort study, the Taiwan Diabetes Study, included all enrollees in the National Diabetes Care Management Program (NDCMP) that has been implemented by the National Health Insurance (NHI) Bureau in Taiwan since 2002.

A population-based cohort study, the Taiwan Diabetes Cohort Study, was conducted during the period 2002 to 2004, including 63,084 ethnic Chinese patients with type 2 DM enrolled in the NDCMP. The aims of NDCMP program were to enhance the quality of diabetes care through intense frequency of monitoring and providing continuity of care to decrease diabetes-related complications. Index date was defined as the date of entry into the NDCMP. All clinically confirmed DM patients were invited to enroll in NDCMP and the diagnosis was based on the criteria of the American Diabetes Association (ADA) (International Classification of Diseases, ninth revision, Clinical Modification (ICD-9-CM) diagnosis code 250).

**FIGURE 1:**
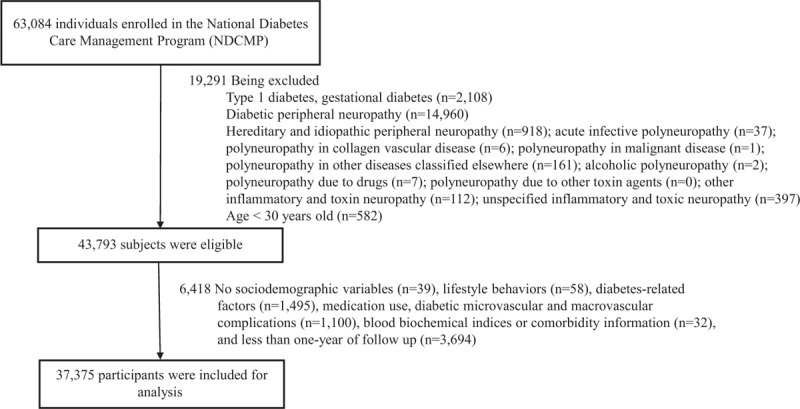
Flow chart of recruitment process of the current study.

For the period 2002 to 2004, the NDCMP initially had 63,084 participants. Patients with type 1 DM (ICD-9-CM Code 250.x1/x3), gestational diabetes (ICD-9-CM Code 648.83), DPN (ICD-9-CM Code of 250.6 or 357.2), other causes of peripheral neuropathy (ICD-9-CM Code of 356.0–356.9, 357.0, 357.1, 357.3–357.9) at baseline, and age <30 years were excluded (Fig. 1). We further excluded the conditions that may influence the accuracy of the diagnosis of peripheral neuropathy at baseline. These conditions include hereditary and idiopathic peripheral neuropathy (ICD: 356.0–356.9; n = 918); acute infective polyneuropathy (ICD: 357.0; n = 37); polyneuropathy in collagen vascular disease (ICD: 357.1; n = 6); polyneuropathy in malignant disease (ICD: 357.3; n = 1); polyneuropathy in other diseases classified elsewhere (ICD: 357.4; n = 161); alcoholic polyneuropathy (ICD: 357.5; n = 2); polyneuropathy due to drugs (ICD: 357.6; n = 7); polyneuropathy due to other toxin agents (ICD: 357.7×; n = 0); other inflammatory and toxin neuropathy (ICD: 357.8×; n = 112); unspecified inflammatory and toxic neuropathy (ICD: 357.9×; n = 397). Those with missing data on socio-demographic variables, lifestyle behaviors, diabetes-related factors, medication use, diabetic micro- and macrovascular complications, blood biochemical indices, comorbidities, and less than 1 year of follow-up from the analysis were also excluded. A total of 37,375 patients were finally included in the present study. The Ethical Review Board of China Medical University Hospital approved the study protocol (CMUH102-REC3-016).

### Data Sources for Baseline and Follow-Up Assessments

In March 1995, the Taiwan government launched the NHI program, which covered approximately 99% of the entire Taiwan population by 1999.^12^ By 2010, the NHI program covered more than 99.62% of the population while the NHI Bureau had contracts with 100% of hospitals and 92% of clinics throughout the nation.^13^ The NDCMP was covered by the NHI program.

The claims data were randomly audited by the insurance system. To enhance the validity of the claims data, quarterly expert reviews on a random sample for every 50 to 100 out-patient and in-patient claims in each hospital and clinic were performed, and the NHI Bureau imposed severe penalties on false diagnostic reports. Previous studies also corroborated the high validity of the data from the NHI program as the primary patient information source for several important population-based studies.^14–17^

In the current study, the datasets for in-patient care by admission and out-patient care for visits from 2002 to 2011 were used. Every individual had a unique personal identification number (PIN). However, data on patient identities in National Health Insurance Research Database (NHIRD) were scrambled cryptographically for security and privacy purposes. Each individual could be interlinked with the PIN in all NHI datasets. The NHIRD data consisted of information on demographic data, date and institution for diagnosis, out-patient visit, in-patient admission, out-patient and in-patient treatment, and physicians providing the services. Due to the comprehensive coverage of the NHI program, the proportion of enrollees withdrawing from it was very low and bias due to loss to follow-up was thus negligible.

Enrollees at the time of entry into the NDCMP underwent a comprehensive health assessment, including systolic blood pressure (SBP), diastolic blood pressure (DBP), their diseases, and complications, together with a series of blood tests, urine tests, and body measurements. All of the participants completed a standardized, computerized questionnaire administered by a case management nurse to record their disease status, medication use, and lifestyle behavior.

Each patient was followed up regularly every 3 to 6 months. On each follow-up year, every patient underwent the same tests as those during entry. After a 12-hour overnight fast, blood was drawn from an antecubital vein in the morning and was sent within 4 hour postcollection for analysis of biomarkers such as HbA1c, fasting plasma glucose, high-density lipoprotein-cholesterol (HDL-C), low-density lipoprotein-cholesterol (LDL-C), triglyceride (TG), and creatinine.

### Outcome Ascertainment

The primary outcome was DPN, which was determined through record linkage with ambulatory and in-patient care data in the NHIRD. The DPN incidence was coded according to the ICD-9-CM as 250.6 or 357.2. All DPN cases in the analysis met at least one of the following criteria: at least 3 ambulatory claims or at least 1 in-patient care claim. The cohort was followed up from the index date to December 31, 2011 or until a DPN event, death, or withdrawal from the national insurance program. By linking the PIN with these computerized files, 8986 newly diagnosed DPN patients were identified in an average of 6.9 years of follow-up.

The status of chronic medical conditions was collected 1 year prior to the index date using out-patient and in-patient claims data. Any history of coronary artery disease (ICD-9-CM codes 410–413, 414.01–414.05, 414.8, and 414.9), congestive heart failure (ICD-9-CM codes 428, 398.91, and 402.x1), stroke (ICD-9-CM codes 430–438), cancer (ICD-9-CM codes 140–149, 150–159, 160–165, 170–175, 179–189, 190–199, 200, 202, 203, 210–213, 215- 229, 235–239, 654.1, 654.10, 654.11, 654.12, 654.13, and 654.14), hyperlipidemia (ICD-9-CM code 272), hypertension (ICD-9-CM codes 401–405), atrial fibrillation (ICD-9-CM code 427.31), chronic hepatitis (ICD-9-CM codes 571, 572.2, 572.3, 572.8, 573.1, 573.2, 573.3, 573.8, and 573.9), chronic obstructive pulmonary disease (ICD-9-CM codes 490–496), and hypoglycemia (ICD-9-CM codes 250, 251.0–251.2) were identified as a comorbidity.

### Statistical Analysis

Baseline HbA1c of each patient was retrieved from datasets of electronic laboratory records and was categorized into 6 levels: <6%, 6 to 7%, 7 to 8%, 8 to 9%, 9 to 10%, and ≥10%. The Lunn–McNeil approach of Cox proportional hazards model, an extended model taking competing risks into account and allowing for multivariate adjustment, was adopted. These extended Cox proportional hazard models with the competing risk of all-cause deaths were used to evaluate the association between HbA1c level and incident DPN.

The hazard ratios (HRs) and their 95% confidence intervals (CIs) by adjusting for age, sex, and traditional variables were calculated. The multivariate model adjusted for age, sex, smoking, alcohol consumption, duration of diabetes, type of hypoglycemic drug (no medication, one oral anti-diabetes drug [OAD], combination of 2, 3, or >3 OADs, insulin monotherapy, and insulin plus OADs), hypertension, anti-hypertensive treatment, overweight (body mass index ≥25 kg/m^2^), elevated blood pressure (SBP ≥130/DBP ≥85 mm Hg), hypertriglyceride (TG ≥150 mg/dL), low HDL-C (HDL-C <40 mg/dL in males; <50 mg/dL in females), high LDL-C (LDL-C ≥100 mg/dL), decreased renal function (estimated glomerular filtration rate, eGFR <60 mL/min/1.73 m), coronary artery disease, congestive heart failure, stroke, cancer, hyperlipidemia, atrial fibrillation, chronic hepatitis, chronic obstructive pulmonary disease, and hypoglycemia. The *P* value was calculated for evaluating the linear trend across the baseline HbA1c categories. The proportionality assumption was tested by including an interaction term for baseline HbA1c categories using follow-up time in the Cox models. There was no statistically significant violation for the proportionality assumption.

The interactions of HbA1c with cardiovascular risk factors at baseline, including elevated blood pressure, hyper-triglyceride, low HDL-C, high LDL-C, decreased eGFR, and number of cardiovascular risk factors, were further examined by adding their product terms into the full model. The likelihood ratio tests were then used to test significance.

The joint effect of HbA1c and each cardiovascular risk factor was explored by creating 3 dummy variables. Using individuals with HbA1c <7% without any cardiovascular risk factor as the reference group, these 3 dummy variables measured the effects of HbA1c ≥7% only, cardiovascular risk factor only, and combined HbA1c ≥7% and any cardiovascular risk factor. All statistical analyses were performed using the SAS software, Version 9.4 (SAS Institute, Cary, NC). Statistical significance was set at a 2-tailed *P* < 0.05.

## RESULTS

During an average of 7.0 years of follow-up, 8379 incidence cases of DPN were identified in patients with type 2 DM, corresponding to a crude incidence rate of 32.04/1000 person-years (30.34 for men and 33.65 for women). Baseline socio-demographic and clinical factors were grouped according to DPN status (Table 1). The DPN patients had a female predominance and had greater mean values of age and diabetes duration. Compared with patients without DPN, those with DPN tended to use insulin injections, use insulin injections plus OAD, have higher prevalence of hyper-triglyceride, and have mean values of the number of cardiovascular risk factors (Table 1).

**TABLE 1:**
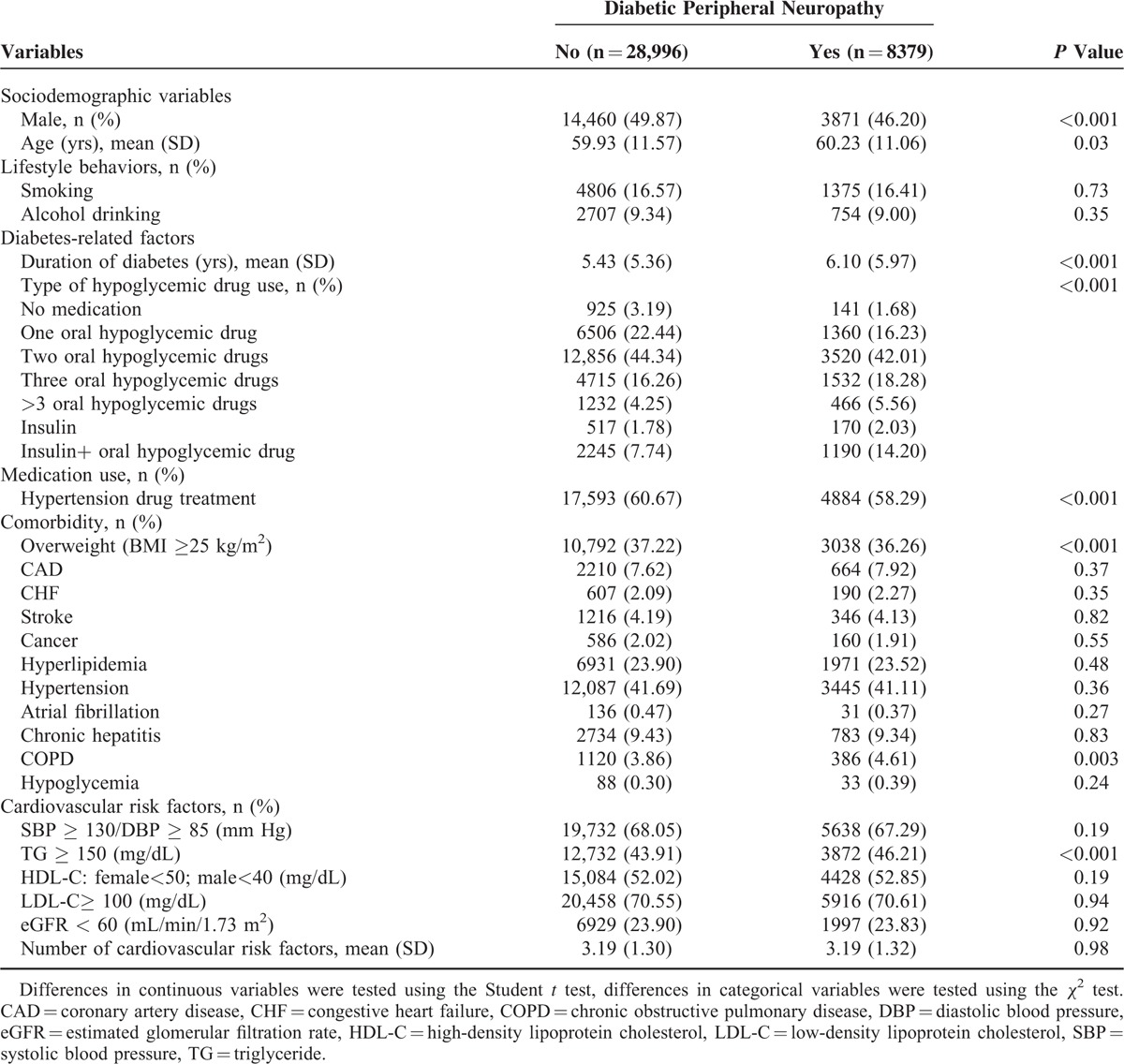
Comparison of Baseline Socio-Demographic Variables, Lifestyle Behavior, Diabetes-Related Factors, Medication Use, Diabetic Micro- and Macro-Vascular Complications, Blood Biochemical Indices, and Comorbidity According to Diabetic Peripheral Neuropathy Incidence in Patients With Type 2 Diabetes Enrolled in the National Diabetes Care Management Program, Taiwan (n = 37,375)

**FIGURE 2:**
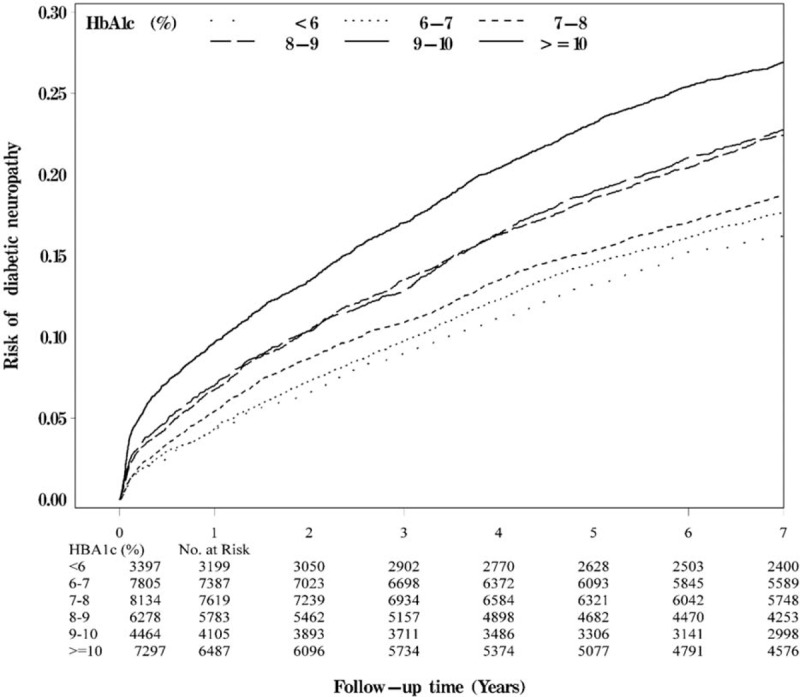
Risks of diabetic peripheral neuropathy by HbA1c level.

The baseline socio-demographic and clinical factors of the patients were grouped according to HbAlc level (Table 2). The Kaplan–Meier cumulative incidence curves of DPN, within the subgroups defined by HbA1c levels, were also presented (Fig. 2). Patients with higher HbA1c levels exhibited a higher risk of developing DPN (*P* < 0.001, by log-rank test).

**TABLE 2:**
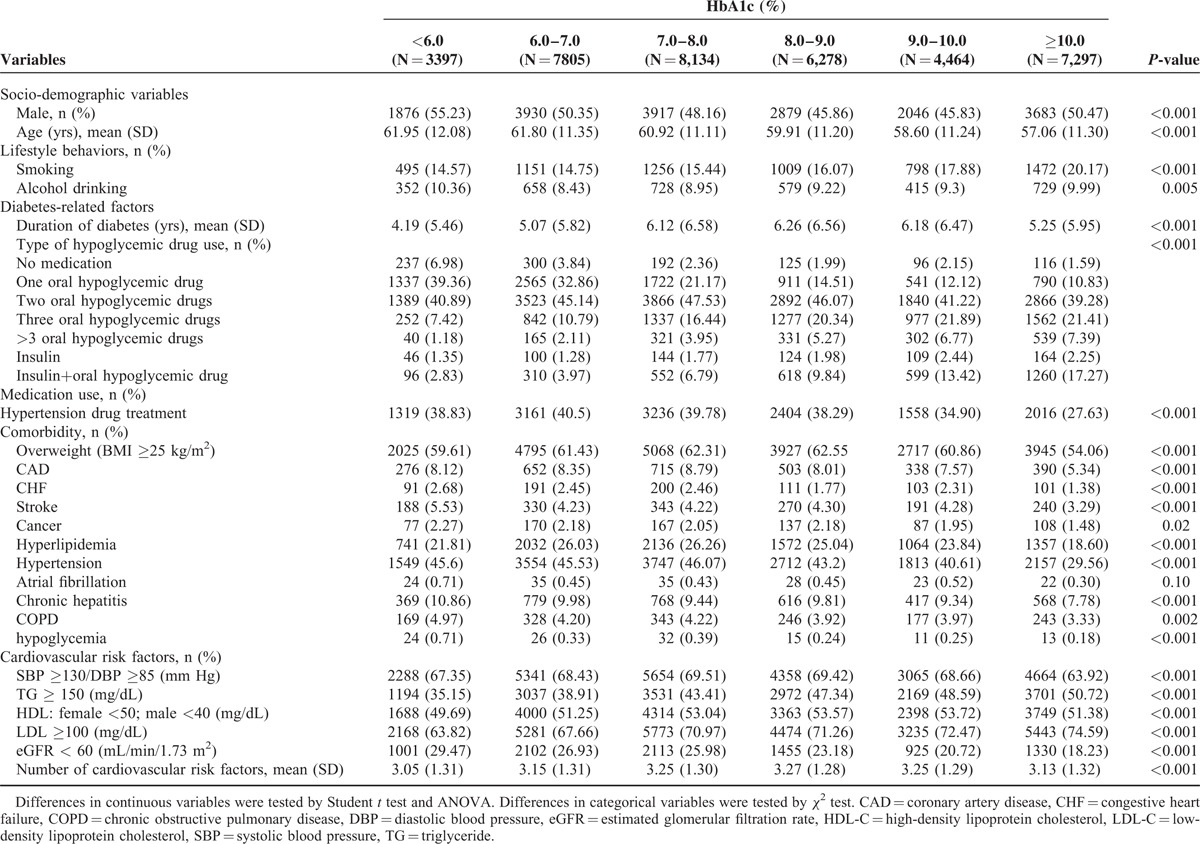
Baseline Socio-Demographic Variables, Lifestyles, Diabetes-Related Factors, Medication Use, and Comorbidity According to HbA1c Level in Type 2 Diabetes Patients Enrolled in National Diabetes Care Management Program, Taiwan (n = 37,375)

The HRs of DPN among the patients were grouped by HbA1c levels (Table 3). Compared with patients with HbA1c level of 6 to 7%, the age-and-sex-adjusted HRs of DPN in patients with HbA1c levels <6%, 7 to 8%, 8 to 9%, 9 to 10%, and ≥10% were 1.22 (95% CI 1.10–1.34), 1.53 (1.42–1.65), 1.91 (1.77–2.06), 2.05 (1.89–2.23), and 2.64 (2.46–2.84), respectively. When considering lifestyle factors, comorbidities, and complications, the effect of HbA1c was slightly attenuated, but remained statistically significant (*P* < 0.05) for those with HbA1c levels 7 to 8%, 8 to 9%, 9 to 10%, and ≥10%. Patients with HbA1c levels 7 to 8%, 8 to 9%, 9 to 10%, and ≥10% exhibited a higher risk of DPN (adjusted HR: 1.11 [1.04–1.20], 1.30 [1.21–1.40], 1.32 [1.22–1.43], and 1.62 [1.51–1.74], respectively) compared with patients with HbA1c level of 6 to 7%.

**TABLE 3:**
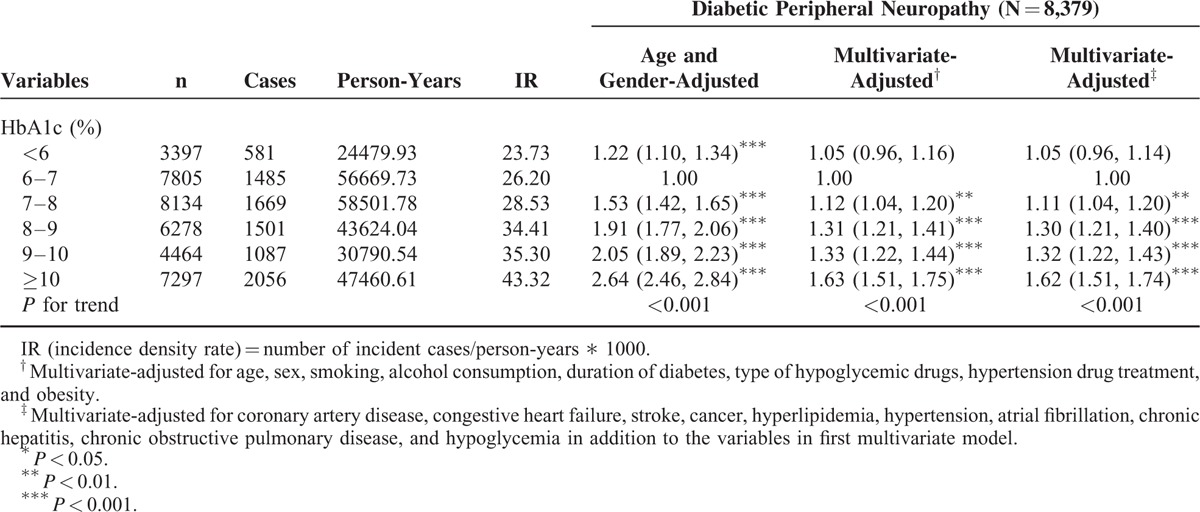
Hazard Ratios (HRs) of Diabetic Peripheral Neuropathy According to Different HbA1c in Diabetic Patients Enrolled in the National Diabetes Care Management Program, Taiwan (n = 37,375)

An increasing trend was observed between the levels of HbA1c and DPN incidence (*P* < 0.001). Sensitivity analyses excluded patients with comorbidities, diabetic ketoacidosis, hyperglycemic hyper-osmolar nonketotic coma, intervertebral disc disorders, diffuse diseases of connective tissue and rheumatoid arthritis, other inflammatory polyarthropathies, and hypoglycemia (n = 36,055 for sensitivity analysis) (Table 4). Similar significant HRs for DPN were found among patients with HbA1c levels of 7 to 8%, 8 to 9%, 9 to 10%, and ≥10% (1.10 [1.03–1.19], 1.29 [1.20–1.39], 1.29 [1.19–1.41], and 1.60 [1.49–1.73], respectively). A sensitivity analysis by including patients aged 30 years old and younger demonstrated the results remain similar and the HRs of DPN for HbA1c levels of 7 to 8%, 8 to 9%, 9 to 10%, and ≥10% were 1.11 [1.03–1.19], 1.30 [1.21–1.40], 1.32 [1.21–1.43], and 1.60 [1.49–1.72], respectively.

**TABLE 4:**
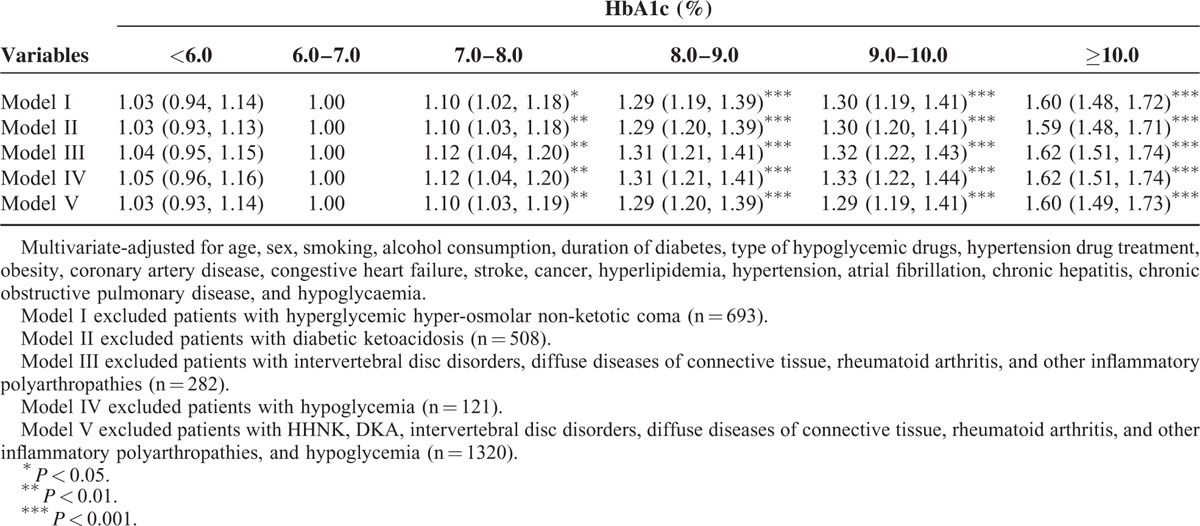
Sensitivity Analyses for Bias Due for Comorbidities After Excluding Patients With Hyperglycemic Hyper-Osmolar Non-Ketotic Coma, Diabetic Ketoacidosis, Intervertebral Disc Disorders, Diffuse Diseases of Connective Tissue and Rheumatoid Arthritis, Other Inflammatory Polyarthropathies, and Hypoglycemia

Further exploring the association between HbA1c level and DPN by stratifying each cardiovascular risk factor (Table 5), the adjusted HRs of DPN for HbA1c level were similar across subgroups of these cardiovascular risk factors. In general, there was a greater magnitude of HR for HbA1c ≥8% as the number of cardiovascular risk factors increased. No significant interactions between HbA1c level and any cardiovascular risk factor were detected (all *P* > 0.05).

**TABLE 5:**
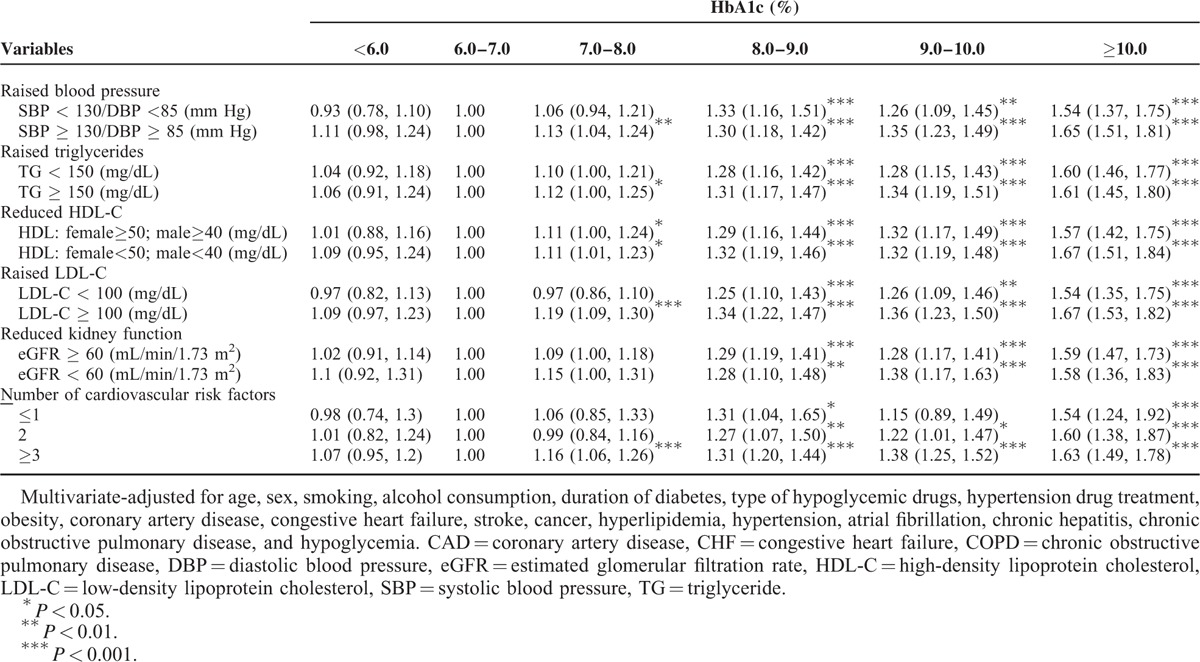
Hazard Ratios (HRs) of Diabetic Peripheral Neuropathy for HbA1c Level According to Each Cardiovascular Risk Factor in Patients With Type 2 Diabetes Enrolled in the National Diabetes Care Management Program, Taiwan (n = 37,375)

**FIGURE 3:**
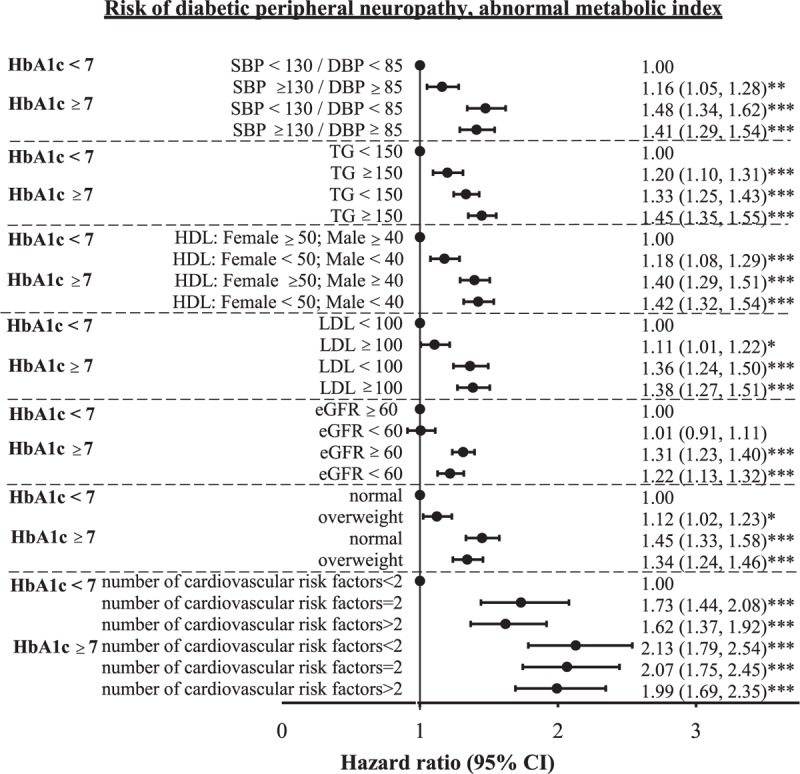
Joint effect of cardiovascular risk factors (e.g., increased blood pressure, hypertriglyceridemia, HDL-C, LDL-C, decreased kidney function, and overweight) and HbA_1_c (<7.0 or ≥7.0) on the risk of diabetic peripheral neuropathy. ^∗^*P* < 0.05; ^∗∗^*P* < 0.01; ^∗∗∗^*P* < 0.001.

The adjusted HR of DPN for HbA1c ≥7% only, each cardiovascular risk factor only, and joint effects of HbA1c ≥7% and each cardiovascular risk factor were determined (Fig. 3). The effects of HbA1c ≥7% were all statistically significant, had narrow 95% CIs, and remained similar ranging from 1.31 [1.23–1.40] when eGFR <60 mL/min/1.73 m^2^ to 1.48 [1.34–1.62] when SBP <130 mm Hg and DBP <85 mm Hg. Cardiovascular risk factors exerting significant independent effect were elevated blood pressure (1.16 [1.05–1.28]), hyper-triglyceridemia (1.20 [1.10–1.31]), low HDL-C (1.18 [1.08–1.29]), high LDL-C (1.11 [1.01–1.22]), overweight (1.12 [1.02–1.23]), and number of cardiovascular risk factors (1.73 [1.44–2.08] for number of cardiovascular risk factors of 2; and 1.62 [1.37–1.92] for number of cardiovascular risk factors >2). The increased DPN risks for joint effects of HbA1c ≥7% with elevated blood pressure, hyper-triglyceridemia, low HDL-C, high LDL-C, decreased eGFR, overweight, and number of cardiovascular risk factors = 2 and >2 were observed when comparing to category of HbA1c <7% and their counterpart reference group (1.41 [1.29–1.54], 1.45 [1.35–1.55], 1.42 [1.32–1.54], 1.38 [1.27–1.51], 1.22 [1.13–1.32], 1.34 [1.24–1.46], 2.07 [1.75–2.45], and 1.99 [1.69–2.35], respectively).

## DISCUSSION

With a follow-up period of 6.9 years, there is an increasing trend between elevated HbA1c and DPN in patients with type 2 DM aged ≥30 years. Cardiovascular risk factors such as elevated blood pressure, hyper-triglyceridemia, low HDL-C, and high LDL-C at baseline appear to be related to newly diagnosed DPN in type 2 DM independent of HbA1c. This finding is consistent after excluding potential confounders in sensitivity analysis, indicating that the study results are robust. After further examining the joint effects of HbA1c level and cardiovascular risk factors, there is a significant joint association between HbA1c ≥7% level and these risk factors, particularly elevated blood pressure, hyper-triglyceridemia, low HDL-C, high LDL-C, decreased eGFR, and number of cardiovascular risk factors with DPN risk. For the aggressive treatment of type 2 DM, as well as other cardiovascular risk factors for DPN prevention, the American Diabetes Association guidelines should be followed.^18^

Elucidating risk factors for DPN is important, given the fact that DPN increases diabetes-related morbidity and mortality.^19,20^ The lack of disease-modifying therapies for DPN also makes the discovery of modifiable risk factors essential.^21^ Evidence points to elevated HbA1c as an independent risk factor for DPN in type 2 DM.^22–24^ The present study confirms previous reports on the strong contributions of elevated HbA1c to the risk of DPN in Chinese patients with type 2 DM. Nonetheless, meta-analyses and multiple trials found that strictly lowering blood glucose alone does not appreciably reduce the incidence of DPN in type 2 DM.^5,6^ The common association of type 2 DM and other cardiovascular risk factors has led to investigations into their effects on DPN.^25^

Using cross-sectional design, 2 independent studies have shown an association between DPN and metabolic syndrome, a combination of cardiovascular risk determinants.^7,8^ Other investigators have shown specific cardiovascular risk factors, including hypertension, obesity, hyper-triglycemia, low HDL-C, and small dense LDL potentially influence the occurrence of DPN.^9–11,24^ However, most of these studies have been done with a small size of sample (n < 1000), short follow-up period, or a cross-sectional study design, which cannot provide a causal relationship between risks factors and DPN or suggest a mechanism that can explain such relationship. In contrast to these prior studies, the strengths of this study include a relatively large number of type 2 DM cases (n > 35,000), the retrospective cohort study design with a sufficiently long follow-up period, and standardized data collection procedures with available information on large numbers of potential confounding factors. The results support the emerging concept that potential cardiovascular risk factors are associated with the risk of developing DPN. These associations suggest a linked disease mechanism, which is worthy of further exploration.

The pathogenesis and underlying mechanisms leading to DPN are complex and not yet fully known.^21,26–28^ Growing experimental studies suggest that although hyperglycemia contributes to the vicious cycles of oxidative stress, inflammation, and nerve damage in type 2 DM, the role of ischemia has also been involved in the pathogenesis of DPN.^26,27^ There is microvascular pathology change in human diabetic polyneuropathy and these vascular changes strongly correlate with clinical deficits and nerve pathology.^27,28^ This is also supported by the reduction of blood flow and endoneurial oxygen tension in the sural nerves of diabetic patients with advanced polyneuropathy.^28,29^ Similarly, there have been a number of animal studies that further strengthen the ischemic/hypoxic mechanisms in the pathogenesis of DPN ^30–35^ Diabetes, hypertension, dyslipedemia, and chronic kidney disease presenting as low eGFR are well documented risk factors for micro- or macroangiopathy.^36,37^ This study suggests that diabetics itself and cardiovascular risk factors identified in this study increase the risk of peripheral nerve ischemia and that this ischemia produces cumulative peripheral nerve injury. Further studies are warranted to elucidate the vascular–metabolic interaction in the pathogenesis of DPN and to conduct clinical trials that target these risk factors as therapeutic strategies for halting or preventing the DPN process.

A growing body of evidence suggests that perturbation of insulin signaling, secondary to insulin resistance, results in neuronal damage and contributes to the pathogenesis of DPN.^38,39^ Decreased expression of insulin-like growth factor and other neurotropic factors in diabetics can lead to decreased repair and perhaps impaired maintenance of peripheral nerve fibers.^40,41^ The risk factors identified in this study, including hypertension, dyslipidemia, low eGFR and insulin resistance, often coexist and hence, drive to cardiovascular disease and DPN.^38,39,42,43^ Since the pathogenesis of DPN is multifactorial, tight glucose control alone will not necessarily reduce dyslipidemia, systemic inflammation, and insulin resistance. Targeting risk factors, as well as lipotoxicity, cellular oxidase, systemic inflammation, and neuronal insulin resistance, will be important in future treatment approaches.

This current study has several limitations. First, the data presented are observational. The possibility of unrecognized residual confounding variables as a potential explanation for findings cannot be excluded, although there are efforts to reduce confounding by statistical adjustment. Second, measurement errors are possible due to the large amount of data gathered from clinical practice. Third, information on DPN subtype is not available. Fourth, only patients with type 2 DM are included in the present study. For the future study, a control group can be included to provide information on increased risk of DPN associated with type 2 DM. In addition, we can evaluate whether the associations between HbA1c as well as cardiovascular risk factors and DPN incidence are modified by type 2 DM status can be evaluated. Fifth, the diagnosis of DPN is primarily clinical and limited clinical information can be obtained from the database. The proper exclusion of other causes of peripheral neuropathy cannot be assured, which may lead to false-positive cases of DPN in these cohorts. However, patients with other common causes of neuropathy have also been excluded in the sensitivity analysis to disprove this possibility.

## CONCLUSIONS

Aside from HbA1c, cardiovascular risk factors, including elevated blood pressure, hyper-triglyceride, low HDL-C, high LDL-C, low eGFR, and number of cardiovascular risk factors, are associated with DPN in type 2 DM patients, suggesting that intensive multifactorial treatment strategies now in use should be rated for their potential to reduce HbA1c level and cardiovascular risk factors in type 2 DM patients to prevent DPN.
